# Supplementary material for: Phenolics Profiling by HPLC-DAD-ESI/MSn of the Scientific Unknown Polygonum hydropiperoides Michx. and Its Antioxidant and Anti-Methicillin-Resistant Staphylococcus aureus Activities
Source: Plants (Basel). 2023 Apr 10;12(8):1606. doi: 10.3390/plants12081606 (PMC10143521; doi:10.3390/plants12081606)
Supplement: Supplementary file 1 [file plants-12-01606-s001.zip › plants-2276276-supplementary.pdf]

Supplementary Materials

Table S1. Identification of phenolic compounds present in the ethyl acetate (EAE-Ph) and ethanolic (EE-Ph) extracts from aerial parts of *Polygonum hydropiperoides*

No.	t_R (min)	[M-H]⁻ *m/z*	*m/z* (% base peak)	Assigned Identification	EAE-Ph	EE-Ph
**1**	2.9	341	MS^2 [341]: 179 (100), 161 (21), 143 (25)<br>MS^3 [341→179]: 161 (46), 143 (49), 119 (100)	Disaccharide	✓	✓
**2**	2.9	331	MS^2 [331]: 271 (36), 211 (17), 169 (100)<br>MS^3 [331→169]: 125 (100)	Galloyl-glucose	✓	✓
**3**	4.0	169	MS^2 [169]: 125 (100)	Gallic acid	✓	✓
**4**	4.2	577	MS^2 [577]: 451 (19), 425 (100), 407 (73), 289 (27), 287 (18)	Procyanidin dimer B-type (epi)catechin–(epi)catechin	✓	✓
**5**	4.7	453	MS^2 [453]: 438 (13), 327 (53), 313 (100), 285 (68), 225 (61), 183 (55), 169 (47)<br>MS^3 [453→313]: 169 (100), 125 (18)	Methylphloroglucinol-*O*-galloyl-glucose		✓
**6**	4.9	577	MS^2 [577]: 451 (26), 425 (100), 407 (63), 289 (32), 287 (8)	Procyanidin dimer B-type (epi)catechin–(epi)catechin	✓	✓
**7**	5.2	431	MS^2 [431]: 385 (100), 179 (44)<br>MS^3 [431→385]: 223 (9), 205 (22), 179 (100), 161 (9)	Hexose derivative		✓
**8**	5.4	289	MS^2 [289]: 245 (100), 205 (31), 203 (20), 179 (20)	Catechin*	✓	✓
**9**	5.9	315	MS^2 [315]: 153 (100), 109 (12)	Dihydroxybenzoic acid hexoside		✓
**10**	6.4	449	MS^2 [449]: 287 (100), 259 (46)<br>MS^3 [449→287]: 259 (100), 243 (8), 125 (6)	Dihydrokaempferol-*O*-hexoside	✓	✓
**11**	8.4	551	MS^2 [551]: 419 (100), 401 (12), 233 (25)<br>MS^3 [551→419]: 404 (100), 377 (33)	Not identified	✓	✓
**12**	8.4	729	MS^2 [729]: 559 (61), 577 (22), 451 (25), 407 (100), 289 (21)	Procyanidin dimer monogallate	✓	✓
**13**	9.7	371	MS^2 [371]: 249 (100)<br>MS^3 [371→249]: 231 (64), 113 (100)	Not identified		✓
**14**	9.8	449	MS^2 [449]: 317 (40), 316 (100)<br>MS^3 [449→316]: 271 (100), 179 (82)	Myricetin-*O*-pentoside	✓	✓

Table S1 (Continuation). Identification of phenolic compounds present in the ethyl acetate (EAE-Ph) and ethanolic (EE-Ph) extracts from aerial parts of *Polygonum hydropiperoides*

No.	t_R (min)	$[M-H]^-$ *m/z*	*m/z* (% base peak)	Assigned Identification	EAE-Ph	EE-Ph
**15**	10.4	463	MS^2 [463]: 317 (100)<br>MS^3 [463→317]: 271 (100), 179 (38), 151 (89)	Myricetin-*O*-deoxyhexoside	✓	✓
**16**	10.9	463	MS^2 [463]: 301 (100)<br>MS^3 [463→301]: 271 (32), 179 (100), 151 (56)	Quercetin-*O*-hexoside	✓	✓
**17**	11.6	441	MS^2 [441]: 331 (16), 289 (100), 169 (47)<br>MS^3 [441→289]: 245 (100), 205 (63), 203 (35), 179 (24), 161 (30)	(Epi)catechin-*O*-gallate	✓	✓
**18**	12.9	623	MS^2 [623]: 315 (100), 300 (24)<br>MS^3 [623→315]: 300 (100), 271 (12)	Isorhamnetin-*O*-rutinoside	✓	✓
**19**	13.5	433	MS^2 [433]: 301 (100)<br>MS^3 [433→301]: 271 (100), 255 (74), 179 (71), 151 (71)	Quercetin-*O*-pentoside	✓	✓
**20**	14.5	477	MS^2 [477]: 301 (100)<br>MS^3 [477→301]: 179 (74), 151 (100)	Quercetin-*O*-glucuronide	✓	✓
**21**	14.9	447	MS^2 [447]: 301 (100)<br>MS^3 [447→301]: 271 (100), 179 (22), 151 (73)	Quercetin-*O*-deoxyhexoside	✓	✓
**22**	15.5	505	MS^2 [505]: 463 (47), 301 (100)<br>MS^3 [505→301]: 271 (71), 179 (56), 151 (100)	Quercetin-*O*-acetylhexoside	✓	✓
**23**	15.9	447	MS^2 [447]: 315 (100), 300 (17)<br>MS^3 [447→315]: 300 (100), 179 (5)	Isorhamnetin-*O*-pentoside	✓	✓
**24**	16.0	537	MS^2 [537]: 491 (100), 341 (28)<br>MS^3 [537→491]: 341 (100)	Not identified	✓	✓
**25**	16.6	781	MS^2 [781]: 735 (100), 271 (9)<br>MS^3 [781→735]: 693 (27), 369 (55), 365 (12), 271 (100)	Not identified	✓	
**26**	17.4	447	MS^2 [447]: 315 (100)<br>MS^3 [447→315]: 300 (11), 271 (92), 285 (100), 243 (28)	Flavonoid-*O*-pentoside	✓	✓
**27**	20.1	461	MS^2 [461]: 315 (98), 314 (100)<br>MS^3 [461→314]: 300 (100), 271 (62)	Isorhamnetin-*O*-deoxyhexoside	✓	✓

Table S1 (Continuation). Identification of phenolic compounds present in the ethyl acetate (EAE-Ph) and ethanolic (EE-Ph) extracts from aerial parts of *Polygonum hydropiperoides*

No.	t_R (min)	$[M-H]^-$ *m/z*	*m/z* (% base peak)	Assigned Identification	EAE-Ph	EE-Ph
**28**	21.0	475	MS^2 [475]: 301 (100)<br>MS^3 [475→301]: 271 (72), 255 (100), 179 (59), 151 (55)	Quercetin-*O*-acetylpentoside	✓	✓
**29**	22.2	563	MS^2 [563]: 169 (100)<br>MS^3 [563→169]: 125 (100)	Gallic acid derivative	✓	✓
**30**	25.3	475	MS^2 [475]: 301 (100)<br>MS^3 [475→301]: 271 (100), 255 (45), 179 (9), 151 (21)	Quercetin-*O*-acetylpentoside	✓	
**31**	27.8	519	MS^2 [519]: 315 (100)<br>MS^3 [519→315]: 300 (100), 271 (10)	Isorhamnetin-*O*-acetylhexoside	✓	
**32**	30.4	331	MS^2 [331]: 316 (100)	Mearnsetin	✓	
**33**	31.1	301	MS^2 [301]: 179 (93), 151 (100)	Quercetin*	✓	✓
**34**	31.5	487	MS^2 [487]: 441 (100)<br>MS^3 [487→441]: 161 (100)	Not identified		✓
**35**	34.9	327	MS^2 [327]: 229 (98), 211 (100), 171 (75)	Oxo-dihydroxy-octadecenoic acid	✓	✓
**36**	35.8	553	MS^2 [553]: 265 (100), 221 (35)	Not identified	✓	
**37**	36.4	779	MS^2 [779]: 633 (100), 487 (20)<br>MS^3 [779→633]: 487 (100), 469 (25), 453 (20)<br>MS^4 [779→633→487]: 307 (48), 235 (38), 163 (100)	Not identified		✓
**38**	37.6	329	MS^2 [329]: 311 (27), 229 (100), 211 (68), 171 (30)	Trihydroxy-octadecenoic acid	✓	✓
**39**	38.2	315	MS^2 [315]: 300 (100), 179 (2)<br>MS^3 [315→300]: 271 (58), 151 (100)	Isorhamnetin	✓	✓

t_R: Retention time; *characterized by comparison with analytical standard

Chromatographic analysis

Following are the chromatograms used for the quantification of the main compounds in the ethyl acetate (EAE-Ph) and ethanolic (EE-Ph) extracts. For compounds numbers, refer to Table 2 (Main manuscript).

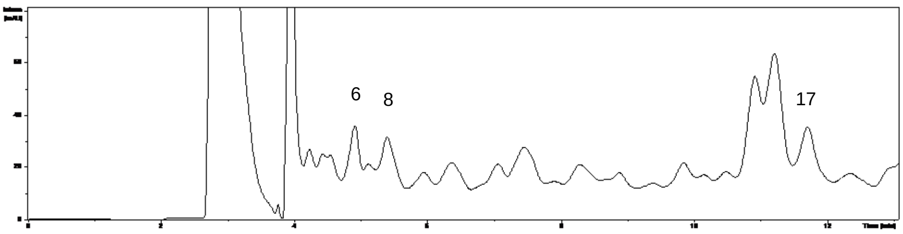

Figure S1. Chromatogram at 280 nm for the ethyl acetate extract (EAE-Ph).

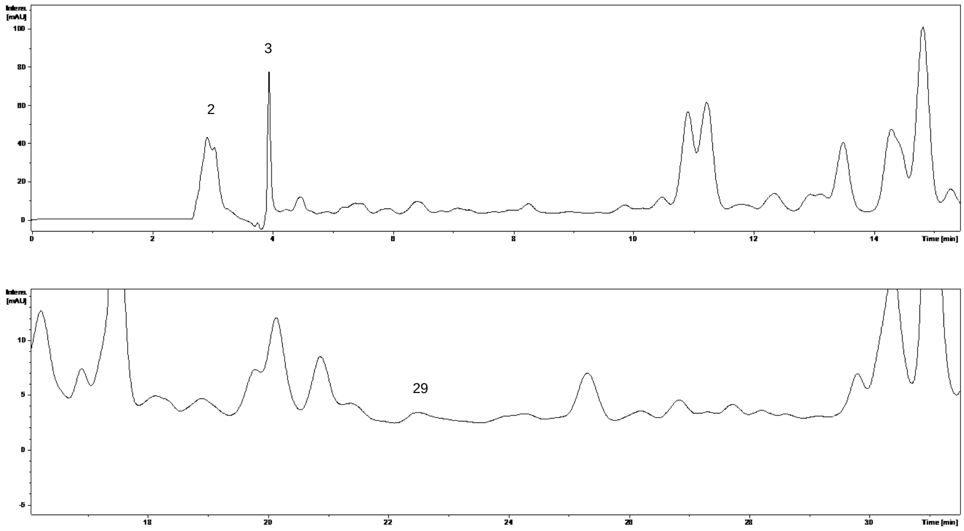

Figure S2. Chromatogram at 320 nm (different zoom depending on the compounds' intensity) for the ethanolic extract (EE-Ph).

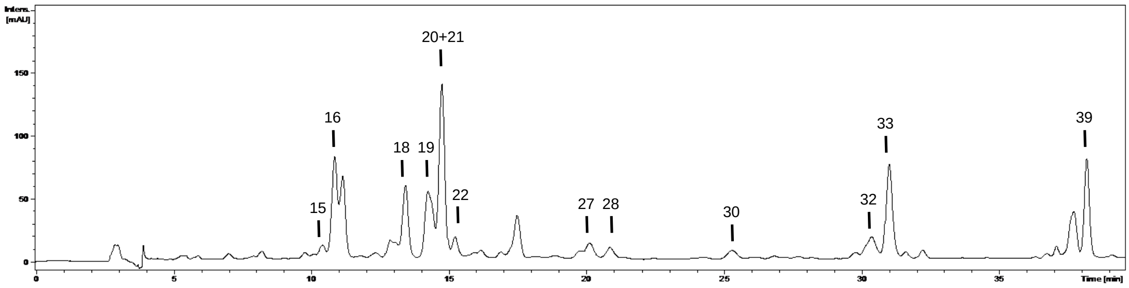

Figure S3. Chromatogram at 350 nm for the ethyl acetate extract (EAE-Ph).

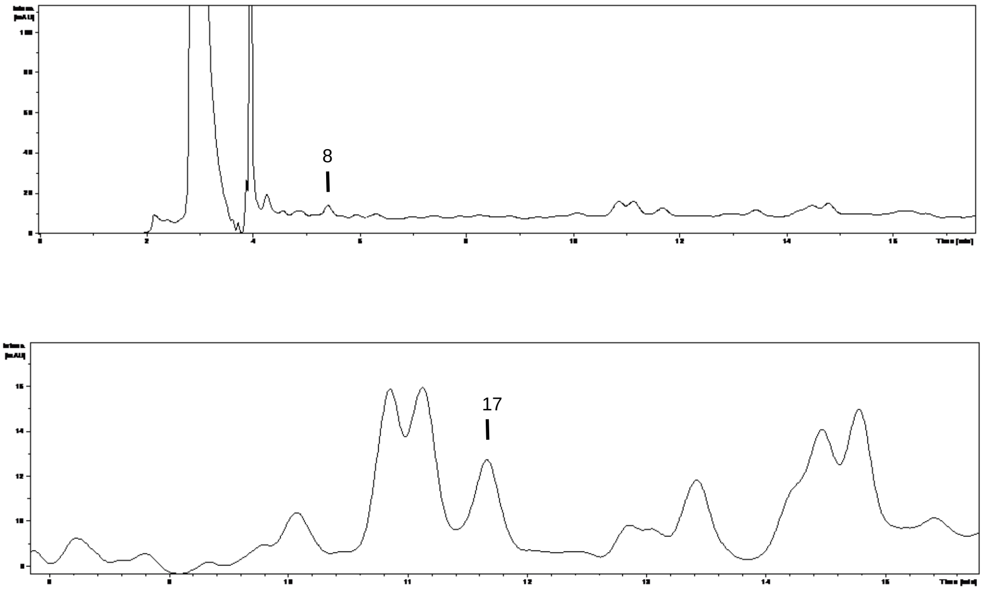

Figure S4. Chromatogram at 280 nm (different zoom depending on the compounds' intensity) for the ethanolic extract (EE-Ph).

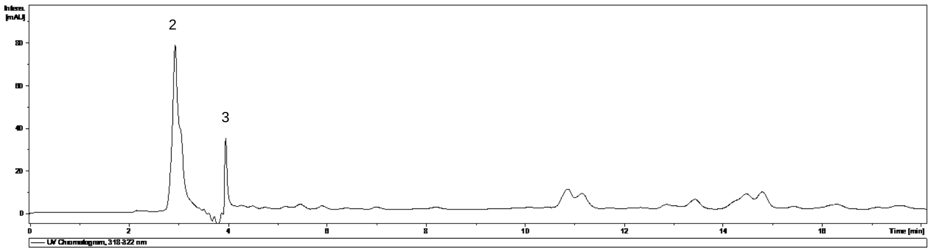

Figure S5. Chromatogram at 320 nm for the ethanolic extract (EE-Ph).

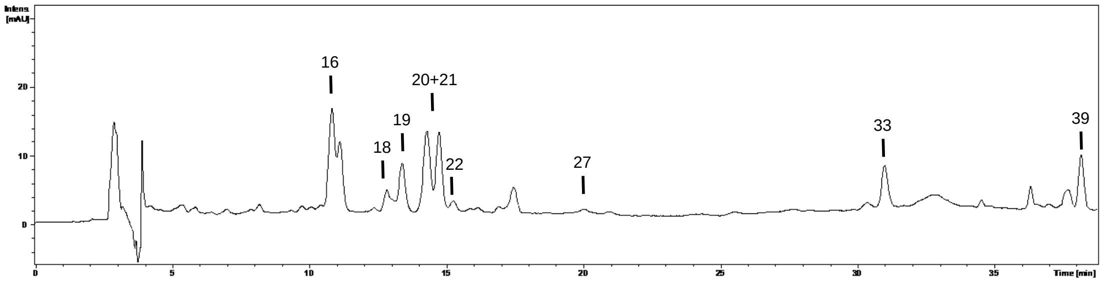

Figure S6. Chromatogram at 350 nm for the ethanolic extract (EE-Ph).
